# Crime and Building Rehabilitation or Demolition: A Dose-Response Analysis

**DOI:** 10.3390/ijerph192013065

**Published:** 2022-10-11

**Authors:** Colette Smirniotis, Michael Henderson, Barbara A. Bailey, Rose M. C. Kagawa

**Affiliations:** 1 Violence Prevention Research Program, Department of Emergency Medicine, School of Medicine, University of California at Davis, Sacramento, CA 95817, USA; 2 California Firearm Violence Research Center, Sacramento, CA 95817, USA; 3 Center on Urban Poverty and Community Development, Jack, Joseph, and Morton Mandel School of Applied Social Sciences, Case Western Reserve University, Cleveland, OH 44106, USA; 4 Department of Mathematics and Statistics, San Diego State University, San Diego, CA 92182, USA

**Keywords:** crime, firearm violence, property demolition, property rehabilitation, spatiotemporal, Bayesian

## Abstract

Thousands of buildings in Cleveland, Ohio were demolished or rehabilitated since the Great Recession in the 2000s. Recent evidence suggests removing vacant and decaying buildings reduces violent and firearm-involved crime. This study examines the dose-response relationship between demolitions, rehabilitations, and crime. We use Bayesian spatiotemporal models to estimate the association of interest for five types of crime outcomes: violent crimes, violent crimes involving a firearm, drug crimes, and crimes often associated with building vacancy. We estimate associations in quarterly time periods from 2012 through 2017 in 569 hexagons approximately the size of a neighborhood (2000 feet, approximately 610 m, in diameter), stratified by vacancy level. Across vacancy levels, the majority of our models do not identify statistically significant associations between demolition and rehabilitation dose and crime incidence. However, in some cases, we identify positive associations between demolition and crime. These associations generally appeared at higher levels of demolition (2 or 3 or more demolitions) in areas characterized by medium to high levels of vacancy. We also find that the presence of a property rehabilitation is associated with an increase in drug crimes in areas with medium levels of vacancy.

## 1. Introduction

Like many cities in the United States, Cleveland, Ohio experienced economic hardship and increasing rates of depopulation following the 2007–2009 Great Recession. Many homeowners lost their homes in foreclosures, partly due to the rise of subprime lending or to economic factors, such as unemployment. The number of vacant and poorly maintained buildings in many neighborhoods grew, further contributing to established trends of population loss and home abandonment [1]. To stabilize local housing markets, local, state, and national agencies worked to demolish or rehabilitate vacant and decaying properties. Building vacancy is associated with increased in crime rates, and recent research suggests building demolition may reduce crime risk [2,3,4,5,6,7]. Less is known about the effects of building rehabilitation on crime or how the association between demolition and crime risk varies across differing levels of demolition. The present study estimates the association between the concentration of demolitions and rehabilitations and the incidence of violent crimes, firearm-involved violent crimes, drug-related crimes, and crimes associated with vacant spaces (e.g., trespassing) in Cleveland neighborhoods from 2012 to 2017.

Criminological theory suggests that criminal activity is partially the rational result of a cost-benefit analysis; an individual balances the potential gain of the crime against the probability and magnitude of punishment [8]. Research has identified vacant buildings as “crime attractors,” features of the environment around which crime tends to occur with greater frequency [2]. Routine activity theory highlights the importance of unguarded spaces, such as vacant buildings, for criminal activity to occur without detection [9].

These theories would suggest removing places that provide opportunities for criminal activity (such as vacant buildings) would reduce crime. Vacant buildings can be removed either through demolition or through rehabilitation and sale. Previous studies of demolition efforts have presented varied results, including a decrease in crime, no significant effect on crime, and a significant, though spatially- or temporally-limited, effect. In Buffalo, New York, one study found that demolitions were associated with lower crime rates at the parcel level (specifically at the site of the demolition), but the magnitude of the association was much smaller when the spatial buffer included the surrounding area (up to 1000 feet, or approximately 305 m); there was no significant effect at the tract level [3]. A study reviewing demolition programs that were a part of the Neighborhood Stabilization Project (NSP) in Cleveland, Chicago, and Denver found that the programs had little to no effect on the number of property or violent crimes when considering the area within 250 feet (approximately 76 m) of a foreclosed property; demolition showed positive but non-significant coefficients for some crime types. The exception was in Cleveland, where they found a small, statistically significant negative association with property crimes in the quarter following demolition, specifically burglary and theft (a decrease of 0.08 crimes per quarter) [4]. A study of demolitions of single-family homes in Saginaw, Michigan found a reduction in violent crime, property crime, and total crime at the block group level in the two months following a demolition [5]. Finally, in Kansas City, Missouri, demolition of vacant buildings was found to have no significant association with crime when comparing the area within 250 feet (76 m) of a demolition to the ring-shaped surrounding area [10].

Though some of the above studies offer evidence in support of building demolition as a crime reduction strategy, less is known about the relationship between demolition dose (how many) and crime risk. One study in Detroit categorized the total number of demolitions in block groups from 2010 to 2014 as low (1–5), moderate (6–10), high (11–20), or very high (21–160), using block groups with no demolitions as the reference. The model measured change in crime counts from 2009 to 2014 and found that demolition was associated with greater reductions in total crime, violent crime, and property crime. The greatest effect sizes were seen in the “very high” demolition block groups, though it was not true that the “low” demolition category always saw the smallest effect on crime [6]. Another study of Detroit’s 2009–2015 demolitions found that block-groups with more than 5 cumulative demolitions by the end of the third quarter of 2015 experienced a significant reduction in firearm assaults but found no significant effect on drug crimes or crime displacement [7].

There are a relatively small number of studies about the effects of building rehabilitation on crime. Rehabilitation of buildings is a broad term that can include small projects to replace broken doors and windows or larger projects to make a building habitable. For example, the Cuyahoga Land Bank, which serves Cleveland, has a set of standards that must be met for their rehabilitated properties; it includes an electric system that meets code, a safe and functional heating system, operable doors and windows, and a roof and foundation that are in good condition [11]. The NSP evaluation in Chicago, Cleveland, and Denver noted that rehabilitation work took many months longer than demolition work, which complicates assessing its effects; longer construction periods allow more time for project-related crime (e.g., theft of building materials), and some studies have found the crime-reducing impact of demolitions to be temporally short-lived [4]. The NSP evaluation included rehabilitation activity, though the number of rehabilitated properties varied greatly between cities. For example, 8.4% of the 1054 Cleveland NSP buildings were rehabilitated, compared to nearly all 139 Denver NSP properties. They found mixed results for the direction of the relationship between rehabilitation activities and crime, concluding that there was no statistically significant relationship [4]. Findings from two studies in Philadelphia, Pennsylvania suggested that property rehabilitation did have a significant effect on crime rates. Specifically, a program that required abandoned buildings to have working doors and windows was associated with decreases in several types of violent crimes (though they also found an increase in property and drug crimes). It should be noted that the type of rehabilitation being studied in Philadelphia was, by design, a quicker, lower-cost version than that found in other programs [12,13].

There is limited research about the quantity of demolition as it relates to crime and about the effect of rehabilitation on crime, especially using robust spatial models. Many prior studies did not account for spatial autocorrelation, and some used open source crime data that anonymized locations by displacing the crime in space by some number of feet, thus introducing greater opportunity for measurement error. In the present study, we seek to add to this body of work by using a Bayesian spatiotemporal model to quantify the association between different concentrations of demolitions and the presence of rehabilitations and the occurrence of violent crime, violent firearm crime, drug crimes, vacant space crimes, and total crimes.

## 2. Materials and Methods

### 2.1. Data

**Figure 1:**
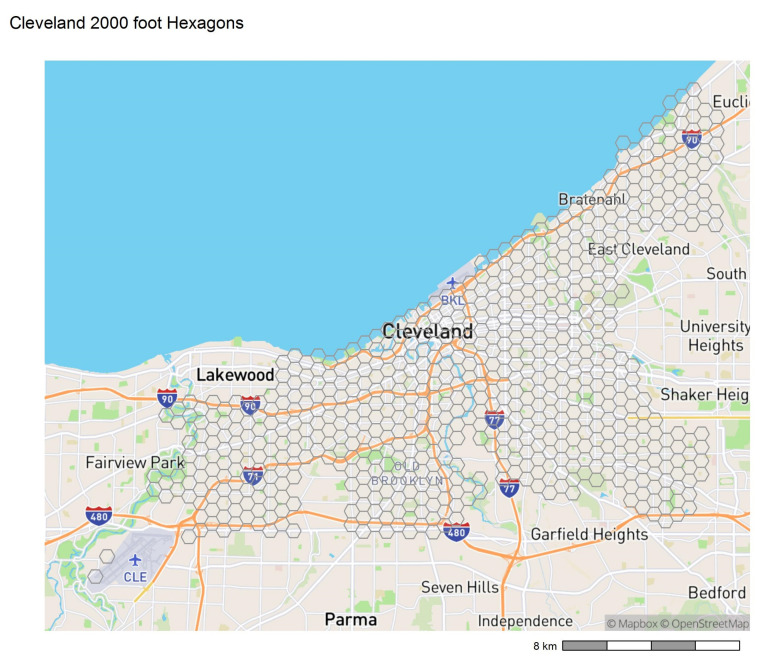
The city of Cleveland, Ohio and the hexagonal grid used in the study.

We divided the city of Cleveland into a regular hexagonal grid of 761 hexagons, each of width 2000 feet (approximately 610 m) (Figure 1) [14,15,16]. This is approximately the size of a superblock (i.e., areas bounded by larger roads or thoroughfares), has the benefit of being uniform in area, and is large enough to allow for variation in the number of demolitions and rehabilitations (treatment “dose”). We excluded hexagons with less than 80% of their area within the city limits and hexagons in the airport neighborhood that did not contain any residential parcel centroids, leaving 569 hexagons for modeling purposes. We used quarterly data from 2012 to 2017 for all variables, unless otherwise noted. Variables from parcel-level data, including demolition, vacancy, and rehabilitation, were aggregated to the hexagon level based on the location of the parcel centroid. There were 11 hexagons that did not contain any parcel centroids; all parcel-related variables were thus interacted with a “zero parcel” indicator, resulting in zero values for those 11 hexagons.

#### 2.1.1. Demolition, Rehabilitation, and Vacancy

We compiled demolition and rehabilitation data from the Cuyahoga Land Bank and the city of Cleveland [17]. Because the distributions of counts of demolitions are highly right-skewed, we converted the quarterly counts of demolition to ordinal variables for each quarter. Demolitions were categorized as low (one demolition per quarter), medium (2), or high (3 or more), with hexagon-quarters receiving zero demolitions as the reference. Rehabilitation of properties was converted from a count to an indicator for each hexagon-quarter because of the large number of zero counts and overall low non-zero counts (third quartile value of zero and maximum value of 6).

For each model, we interacted demolition and rehabilitation with vacancy in order to estimate effects separately for low, medium, and high vacancy areas. The distribution of counts of vacancies was highly right-skewed. Vacancy was converted to categories of low (0–1 vacancies), medium (2–11), and high (12 or more). To ensure the proper time ordering of events within a quarter, demolition, rehabilitation, and vacancy values were lagged with respect to crime outcomes; as a result, our model begins in the second quarter (Q2) of 2012.

#### 2.1.2. Crime Outcomes

We estimated effects for five crime outcomes: Federal Bureau of Investigations Part I violent crimes defined per the Uniform Crime Reporting handbook (homicide, rape, aggravated assault, and robbery), referred to here simply as “violent crime”; Part I violent crimes with a firearm; drug crimes; crimes associated with building vacancy, referred to here as “vacant space” crimes (arson, theft from a building, destruction of property/vandalism, prostitution, disorderly conduct, trespassing, and curfew/loitering/vagrancy violations); and total crime (the sum of violent crimes, drug crimes, and vacant space crimes) [18]. Crime data from the Cleveland Police Department spanned 2012 Q2 to 2017 Q4, though due to a change in reporting practices, the counts of firearm versus non-firearm related violent crimes in 2016 and 2017 were inconsistent with previous years [19]. Our analysis only used data through 2015 for the violent crimes with a firearm outcome. The total count of violent crimes was not affected. Although hexagons that had greater than 20% of their area outside the city boundary were removed, some hexagons still crossed city boundaries. Crime data were not available in areas outside of Cleveland, which could lead to under-reporting of outcomes in hexagons along the city border. To estimate a correction, we multiplied the number of observed crimes in partial hexagons by the inverse of the proportion of their area located inside the city limits. For modeling purposes, the crime counts were rounded to the nearest integer when used as the outcome of interest.

#### 2.1.3. Control Variables

Control variables were lagged by one quarter with respect to the outcome crime, as was done with demolition, vacancy, and rehabilitation. We used 46 property-related variables, including counts of buildings, vacant lots, dilapidated buildings, and types of buildings (e.g., industrial, residential, commercial); median sales price per square foot; and percentages of tax- and mortgage-foreclosed parcels for 2012 Q2 through 2017 Q4. These values were drawn from the Cuyahoga County Fiscal Office records using the Northeast Ohio Community and Neighborhood Data for Organizing (NEOCANDO) data system [17]. Each value was reported quarterly, except the annually reported counts of dilapidated buildings, which we repeat for each quarter in the given year. Where appropriate, variables were interacted with indicators identifying when a characteristic did not apply to the hexagon. For example, the variable mean number of bathrooms per residence was interacted with an indicator of the presence of at least one living space in the hexagon. There was a small amount of missing data for two variables in 10 hexagons, for which we conducted multiple imputation, as described further in Appendix A.1. To control for potential confounding effects of historical property remediation efforts, we used data from 2010 Q1 forward to create four variables that capture the number of previous demolitions and rehabilitations that occurred in a hexagon: the sum of demolitions in the four previous quarters and the sum in the four before that, and an indicator variable of rehabilitation in the four previous quarters, and the four before that.

We included 16 community demographic variables to capture neighborhood characteristics, including unemployment, household income, education level, age, and race of residents in each hexagon. These data were sourced from the American Community Survey rolling five-year estimates for block groups and applied to hexagons using population weighted averages [20]. We included a quarterly weighted average number of employees per hexagon, estimated from the census tract values in Longitudinal Employer-Household Dynamics data from the Census Bureau [21].

Additional measures of crime associated with the outcomes of interest were used as explanatory variables [19]. See Appendix A.2 for details.

Since many of these variables are highly correlated, we used principal component analysis (PCA) to reduce dimensionality. PCA was performed separately on each set of property, community, and crime predictor variables for each outcome, and the first principal component of each was then used in the corresponding model. The exception to this was the model for total crimes, for which we omitted the crime principal component due to overlap in outcome and predictor crimes. Finally, we included quarterly indicator variables to account for the seasonality of crime.

### 2.2. Model

Using a Bayesian hierarchical spatiotemporal framework featuring conditional autoregressive priors, we modeled crime counts by type with demolition, property condition, crime, and community demographic covariates. Specifically, we used a Poisson generalized linear mixed model, in which the spatial surface can vary over time, with the random effects in each time period following an autoregressive process of order 1. A conditional autoregressive prior for the precision matrix controls the spatial autocorrelation. This model controls for two common features exhibited in our data: temporal correlation, stemming from the similar population and conditions in successive quarters; and spatially-correlated data, arising from neighborhood effects and/or unobserved covariates [22].

The model specifications are as follows. The 569 non-overlapping hexagonal units are denoted as areal units $S_{k}$ where k=1,2,⋯,K and *K* = 569, and the study covers consecutive time periods t=1,2,⋯,N. The crime outcome of interest is a vector of the count of crimes in each of the *K* hexagons $Y={\left(Y_{1},\cdots ,Y_{N}\right)}_{K\times N}$, and $Y_{t}=\left(Y_{1t},\cdots Y_{Kt}\right)$. The *p* regression parameters are denoted as $\beta =(\beta_{1},\cdots ,\beta_{p})$, and the observations are denoted as $x_{kt}=\left(x_{kt1},\cdots ,x_{ktp}\right)$ for areal unit *k* at time period *t*. $O_{kt}$ is a vector of known offsets; this term in our setting is trivial, as we are concerned about crime counts per hexagon, with each hexagon having equal area. The adjacency matrix $W_{K\times K}$ is a binary matrix with $w_{kj}=1$ if hexagons *k* and *j* are neighbors [22,23].

(1) $$Y_{kt}∼Poisson\left(\mu_{kt}\right)$$

(2) $$\ln\left(\mu_{kt}\right)=x_{kt}^{T}\beta +O_{kt}+\psi_{kt}.$$

(3) $$\beta ∼N(\mu_{\beta },\Sigma_{\beta })$$

(4) $$\psi_{kt}=\phi_{kt}$$

(5) $$\phi_{t}|\phi_{t-1}∼N\left(\rho_{T}\phi_{t-1},\tau^{2}Q{\left(W,\rho_{S}\right)}^{-1}\right)$$

(6) $$\phi_{1}∼N\left(0,\tau^{2}Q{\left(W,\rho_{S}\right)}^{-1}\right)$$

(7) $$\tau^{2}∼\mathrm{Inverse}-\mathrm{Gamma}\left(a,b\right)$$

(8) $$\rho_{S},\rho_{T}∼\mathrm{Uniform}\left(0,1\right)$$

The model and Markov chain Monte Carlo simulations were implemented in R via the CARBayesST package, using the ST.CARar function to model crime counts with a Poisson distribution [22,24,25]. We created three independent Markov chains for each outcome, each of size 4,200,000, with a burn-in period of 200,000 samples and thinned by 1000, resulting in a final combined MCMC sample of size 12,000. We estimated the model parameters (median) and calculated a 95% credible interval for the parameter estimates using quantiles (0.025 and 0.975). We generated the posterior distribution of the linear combination of covariates, and then used the quantiles to estimate the effect of demolition and rehabilitation across vacancy and demolition groups. By exponentiating these quantiles of posterior distributions, we calculated an estimate and credible interval for the relative risk for a 1-unit increase in each of demolition and rehabilitation.

## 3. Results

### 3.1. Descriptive Statistics

Over the course of the study period, 6505 properties were demolished. The demolitions occurred in 3408 hexagon-quarters (396 distinct hexagons), yielding a mean of 1.909 and a maximum of 13 demolitions per hexagon-quarter in demolition-treated hexagons. Building rehabilitation occurred in 6.9% of hexagon-quarters, with a mean of 1.152 and a maximum of 6 rehabilitations per hexagon-quarter in rehabilitation-treated hexagons.

There was a total of 30,490 violent crimes (mean 2.330 and maximum 24 per hexagon-quarter), 6762 violent crimes with a firearm (mean 0.792, maximum 11), 13,675 drug crimes (mean 1.045, maximum 41), 66,231 vacant space crimes (mean 5.061, maximum 69), and 110,396 total crimes (mean 8.436, maximum 114) over the study period.

The mean number of parcels per hexagon-quarter was 271, and the mean number of major buildings per hexagon-quarter was 224. Hexagons contained high numbers of parcels with single family residences (mean of 142 parcels per hexagon-quarter) and small (two- to four-unit) apartment buildings (mean 51) (Table 1). The mean population per hexagon-quarter was 647 people, with the following means of demographic proportions per hexagon-quarter: 19.6% under the age of 18, 42.3% male, 31.3% below the poverty line, and 16.1% unemployed. Summary statistics for the additional variables can be found in Appendix B (Table A1 and Table A2).

**Figure 2:**
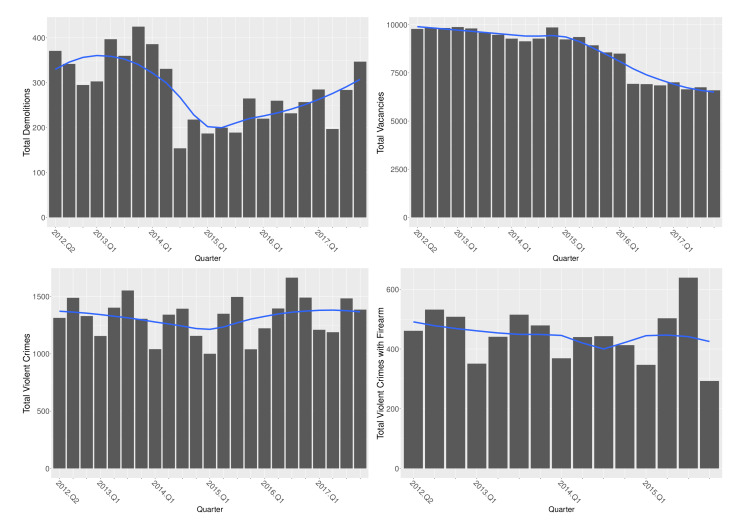

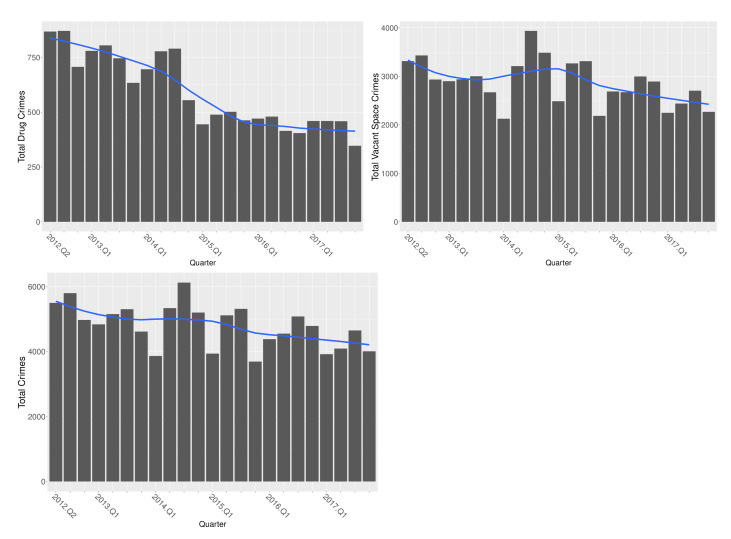
Citywide quarterly total demolition, vacancy, and crime counts over study duration, with LOESS smoother.

Considering the citywide temporal trend for demolitions, vacancies, and crimes from 2012 to 2017 (Figure 2), demolitions abruptly decreased in the middle of the study period and slowly increased thereafter. Total vacancies decreased over time but held steady for the last two years. Violent crimes (total and with firearm) appeared relatively stable over time. Drug and vacant space crimes decreased over the study period, with drug crimes decreasing at the fastest rate. 

**Figure 3:**
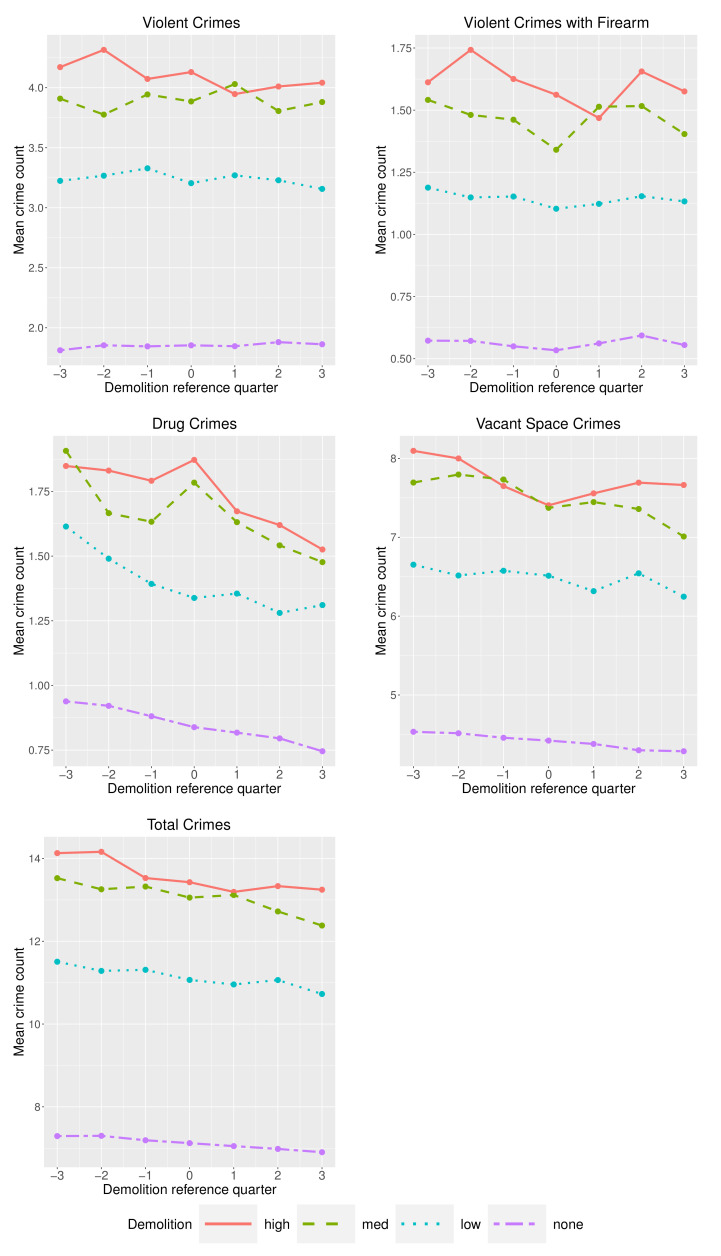
Mean crime counts in the three quarters before and three quarters after demolition(s).

We explored the overall trend in crime counts in hexagons before and after a demolition. Within each demolition group, we calculated the mean quarterly number of crimes in a hexagon starting three quarters prior to a demolition and ending three quarters after a demolition, following the example of Stacy [5]. For violent crimes (total and firearm), the overall trend of the means is relatively flat (Figure 3). Drug crimes show a decrease at all levels of demolition, though they show an increase in the demolition quarter for medium and high demolition levels. Vacant space crimes show a general downward trend across all demolition levels, though in hexagons with a high level of demolition, vacant space crimes increase slightly following demolition. Total crimes in all hexagons decrease at approximately the same rate before and after demolition, regardless of the level of demolition. The intercepts, however, differed notably across demolition level with greater numbers of demolitions received in neighborhoods with higher crime counts.

**Figure 4:**
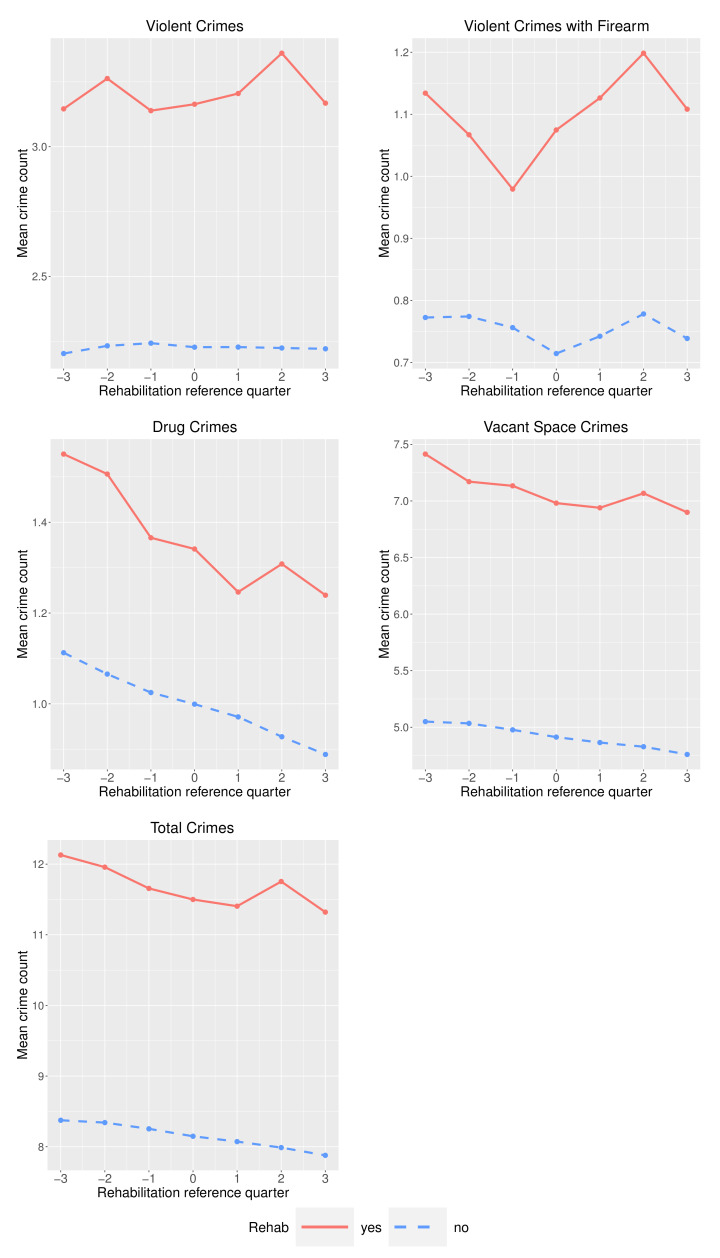
Mean crime counts in the three quarters before and three quarters after rehabilitation(s).

We also explored the overall trend in hexagons before and after a rehabilitation using the same approach [5]. For all crime types, the mean crime counts are higher for the hexagons that received property rehabilitation (Figure 4). Average counts of violent, drug, vacant space, and total crimes appear to follow similar pre-treatment trends in hexagons that did and did not receive rehabilitation. However, in all five cases, the average count increases in the second quarter following a rehabilitation. For violent crimes with a firearm, both groups show an increase in mean crime count two quarters after the rehabilitation. It should be noted that for the rehabilitation group, the mean number of violent crimes involving a firearm was decreasing for two quarters with the increasing trend starting one quarter prior to the rehabilitation. Again we see similar decreasing trends in drug crimes, vacant space crimes, and total crimes, regardless of presence or absence of rehabilitation and despite higher crime in hexagons that received rehabilitation.

**Figure 5:**
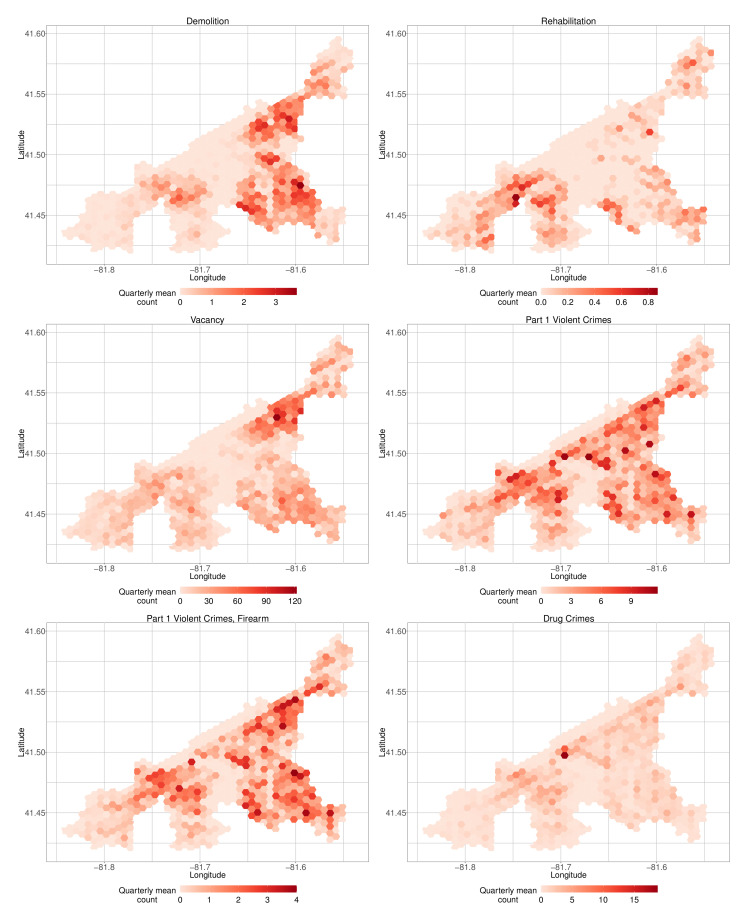

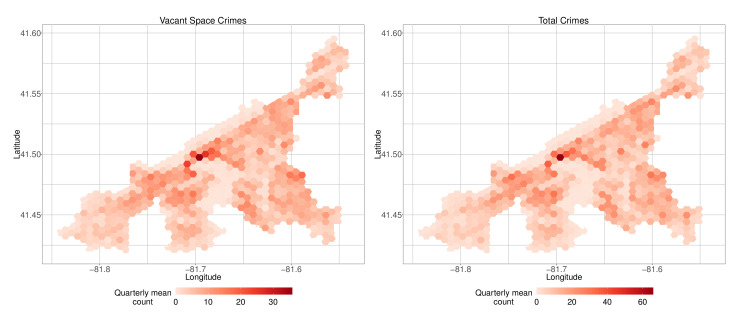
Mean quarterly demolition, vacancy, and crime counts per hexagon.

This framing of the crime counts as “before and after” demolition does not control for the potentially confounding effects of other variables or account for spatial autocorrelation. Our outcomes demonstrate positive spatial autocorrelation via a permutation test for Moran’s I (*p*-values less than 0.0001). The mean quarterly counts of each of demolition, vacancy, and crime counts in the city (Figure 5) also demonstrate this strong spatial pattern. For example, hexagons with high mean vacancy counts and high mean demolition counts overlap with areas with high violent crimes. It is, therefore, critical to account for spatial autocorrelation in the modeling strategy.

### 3.2. Model Results

**Figure 6:**
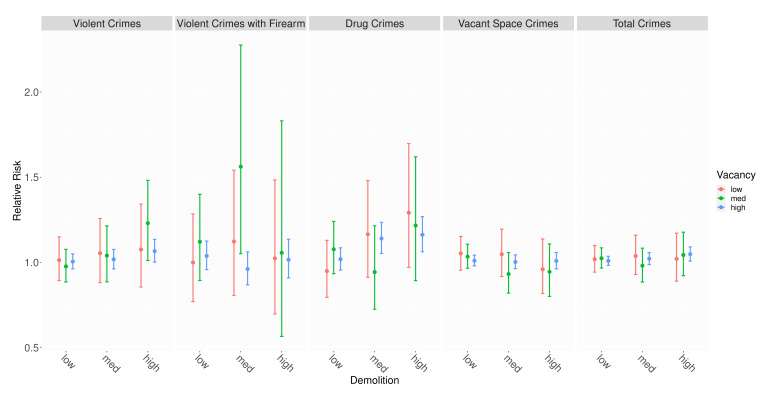
Relative risk of demolition (posterior median and 95% credible interval) by crime outcome.

The relative risk for each level of demolition compared with no demolition within vacancy group is shown in Table 2 and Figure 6. The model parameters are available in Appendix C, Table A3 and Table A4. Almost all credible intervals for relative risk include 1, indicating no significant association between demolition and crime, regardless of dose. However, there are six intervals that suggest a positive association between demolition and the incidence of crime: violent crimes in hexagons with medium or high vacancy and high demolition; violent firearm crimes in hexagons with medium and medium demolition; drug crimes in hexagons with high vacancy and medium or high demolition; and total crimes in hexagons with high vacancy and high demolition. Additionally, the magnitude of the associations with violent crimes and drug crimes increases at higher demolition doses, though that relationship does not hold for the other crime types.

**Figure 7:**
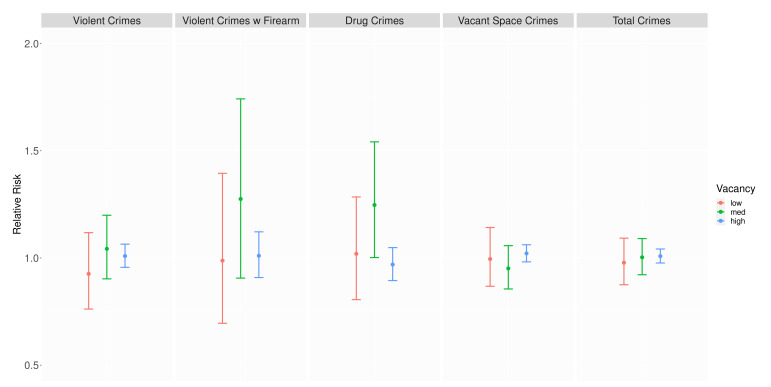
Relative risk of rehabilitation (posterior median and 95% credible interval) by crime outcome.

Using the same approach of linear combinations of posterior median and credible intervals, we estimate that the presence of a rehabilitated property does not have a significant association with crime at any level of vacancy, with one exception. We find a positive association between rehabilitation and drug crimes in hexagons with medium levels of vacancy (Table 3 and Figure 7).

## 4. Discussion

The current study analyzed the dose-response relationship between property demolitions and crime and the relationship between property rehabilitation and crime in Cleveland, Ohio from 2012 through 2017. Using quarterly data and a Bayesian spatiotemporal framework, this study found that neither property demolitions nor rehabilitations were associated with a decrease in the incidence of nearby crime in the following quarter and, in some cases, were associated with an increase in the incidence of crime. Increases did not display consistent patterns across demolition dose, vacancy level, or crime type. However, associations generally appeared at higher levels of demolition (2 or 3 or more demolitions) in areas characterized by medium to high levels of vacancy.

The modeling approach presented here included several novel features to add to the existing body of work on the topic. First, our Bayesian model accounted for spatial, temporal, and spatiotemporal autocorrelation. Previous studies on this topic often address spatial autocorrelation using frequentist models and a spatial lag or not at all. Controlling for space and time in the context of property remediation is particularly important because these remediation efforts, and the factors associated with these efforts, such as poverty, unemployment, and foreclosure rates, are often concentrated in space and across time and may confound associations of interest. Second, few studies have explored whether there is a dose-response relationship between property remediation efforts and crime. It is valuable to understand what concentration (i.e., the number of vacant and decaying buildings demolished or rehabilitated) is needed to achieve the desired level of change. The effects of property rehabilitation on crime is also understudied relative to property demolition, yet we had hypothesized rehabilitation would yield greater crime reduction effects stemming from the positive investment in a place, the addition to “eyes on the street” that a newly occupied building brings, and the reduction in unguarded spaces. Finally, we tested effects stratified by housing vacancy levels to test whether the effectiveness of property remediation as a crime prevention tool varied by community characteristics.

The effects of demolition on crime likely vary across the size of spatial unit and the time over which effects are estimated. The current study contributes evidence of the effectiveness of demolition and rehabilitation in areal units 2000 feet in diameter and in the quarter following demolition activity. Using a microplace analysis can miss effects happening at a larger spatial unit, but neighborhoods can simultaneously be both too large and too small a unit to use. If the unit of analysis is “too large,” it falsely assumes homogeneity, and if it is “too small,” it ignores factors happening outside of or adjacent to the neighborhood [26]. We selected hexagons of size 2000 feet to maximize variability in demolition dosage. However, this limits our ability to observe associations that might occur at a more micro-level. Previous studies have found protective associations with demolition proximal to the demolition site (at the parcel or within 350 to 700 feet) [3,27]. Although our modeling decisions sought to maximize opportunities for observing effects by exploring large doses over a relatively short time interval, our geographic unit may have been too large to identify the effects reported elsewhere [5,6,7].

Selecting a time period is similarly challenging. Some studies have found demolition to have an effect on crime for a time period shorter than a quarter, but property rehabilitation can take on the order of months [4,5]. Two studies that considered demolition from a dose-response perspective found statistically significant reductions in crime in areas with larger amounts of demolition; however, in these cases, associations were measured over a much longer period of time (on the order of years) than in the present study [6,7]. Our results may depart from previous studies that found demolition had either a protective effect on violent or drug crimes or no effect [3,4,5,6] if it is the case that demolitions are followed by short-term (1–3 months) increases in crime that later decrease.

Prior research suggests the demolition or rehabilitation of vacant and deteriorating or dangerous buildings may affect crime by removing physical deterrents to social interaction or repopulating areas, improving the potential for guardianship, removing hidden spaces that facilitate crime, and strengthening social organization. A recent study of Philadelphia, Pennsylvania (2008–2018) illuminated these ideas by finding that only privately-funded demolitions (not publicly-funded or the combination of both) were associated with a reduction in crime. The authors suggested this was due to two factors—publicly-funded demolitions tended to occur in neighborhoods that had experienced more extreme economic decline, and privately-funded demolitions were seen as a signal of meaningful community investment [28]. The distinction between types of demolitions was not included in the current study but could be considered for future work.

For rehabilitation, we find evidence for an increase in drug crimes, only in areas with medium levels of vacancy. Finding no significant reduction in violent or property crimes, or even a slight increase, associated with rehabilitation of properties is in line with other research [4]. This result was an outlier (i.e., the only of 15 tests to suggest a departure from natural variation) in the current study, and while it does not depart from findings from previous studies, future research is needed to help illuminate the relationship between property rehabilitation and crime across other geographies and time horizons.

This study is subject to a number of limitations. It is possible that using PCA for control variables as described above does not fully control for the confounding effects of the individual variables involved. The location of crimes may have been geocoded to the nearest intersection, resulting in some misclassification when assigned to hexagons. Further, it is possible that the associations produced from our models capture the effect of crime on demolition (i.e., reverse causation). Demolition efforts may specifically target neighborhoods with higher levels of crime, and while we have included time lags to reduce the effects of bidirectional causation, we may not have completely removed this association. Finally, it is difficult to account for dispersal of crime versus a true reduction in crime. For example, a study of the spatial relationship of demolition and crime (assault, drug, and prostitution) using a cluster analysis in Buffalo, New York found that while crime decreased in the demolition zone, criminal activity shifted away from that area, creating clusters of crimes in other parts of the city [29]. The models presented in this study do not measure dispersion directly.

The present study contributes information about associations between crime and property demolition and rehabilitation over relatively short time periods (1–3 months) and within relatively large neighborhood units and does not find a consistent association. These results evaluating the effectiveness of property demolitions and rehabilitations, and others like it, can be of particular importance to local government entities challenged by demographic and residential trends that result in vast swaths of unused, decaying housing, as well as to groups focused on crime and firearm violence prevention. There are very few (if any) single policy levers powerful enough to make a meaningful difference against longstanding, systemic problems, such as concentrated poverty and the chronic disinvestment in central cities. Specific policies, such as demolition, may yield crime reduction benefits over specific time periods and distances. More broadly, making real headway against these problems and their outgrowths, such as crime and gun violence, depends on sustained, significant, and coordinated actions involving multiple policy organizations [30,31].

## 5. Conclusions

Some recent studies have suggested that programs to demolish or rehabilitate properties can reduce firearm violence and other crime. However, using a spatiotemporal model, we do not find that demolition and rehabilitation are associated with a decrease in firearm violence in Cleveland during our study period. These null results, and the results indicating an association with an increase in crime in some select cases, add to the body of work that has generally identified more protective associations between demolition and crime over some time periods and across some geographic units. Taken together, this work suggests these programs have limitations as crime prevention measures and should be coupled with other forms of investment and prevention. It is not a coincidence that crime and vacancies are frequently co-located; a history of social and geopolitical decision-making, influenced explicitly and implicitly by racism, defines the geographic distribution of opportunity and poverty in the United States. The demolition and rehabilitation of properties has limited impact on these structural drivers of violence, especially in the short term, and, as such, may have limited potential as crime prevention strategies without linking demolition and rehabilitation with greater investments in (and in partnership with) communities.

## Figures and Tables

**Table 1:** Mean (standard deviation) and median of hexagon-quarters (hex-qtrs).

Hex-qtrs with Parcel Centroids (*N* = 12,834)	Mean (St Dev)	Median
Parcels (count)	271.305 (168.036)	277.500
Living units (count)	301.540 (224.995)	295.000
Single family homes (count)	142.491 (121.67)	118.000
Small multi-unit homes (count)	51.221 (57.335)	31.000
Large multi-unit homes (count)	2.431 (3.781)	1.000
Condos (count)	0.150 (0.554)	0.000
Commercial buildings (count)	8.896 (11.721)	5.000
Industrial buildings (count)	3.496 (5.481)	1.000
Vacant lots (count)	55.000 (56.263)	35.000
Lot square footage (median)	48,915.949 (364,749.286)	5119.000
Buildings (count)	224.107 (151.462)	217.000
Buildings (proportion)	0.894 (0.308)	1.000
Residential sales past 2 years (proportion)	0.830 (0.376)	1.000
Residential sales past 3 years (proportion)	0.842 (0.365)	1.000
Residential sales past 5 years (proportion)	0.854 (0.353)	1.000
Land Bank owned (count)	21.135 (33.78)	5.000
Tax delinquent (proportion)	0.100 (0.086)	0.078
**Hex-qtrs with buildings (** ***N*** **= 11,468)**		
Building age (median)	85.933 (24.209)	92.000
Effective age of building (median)	55.858 (12.104)	57.100
Vacant buildings (proportion)	0.072 (0.071)	0.059
Vacant buildings (count)	17.270 (18.094)	12.000
Occupied units (proportion)	0.995 (0.071)	1.000
Number of property transfers (mean)	0.490 (0.267)	0.480
Number of arms length transfers (mean)	0.191 (0.113)	0.186
Property transfers (count)	91.859 (52.573)	90.000
Building square footage (median)	1477.968 (406.039)	1462.000
Brick (proportion)	0.148 (0.212)	0.074
Bathrooms (mean)	1.556 (0.373)	1.561
Bedrooms (mean)	3.579 (0.696)	3.609
Tax delinquent units (proportion)	0.168 (0.141)	0.144
**Hex-qtrs with residential sales in 2/3/5 years (** ***N*** **= 10,660/10,811/10,971)**		
Residential sales past 2 yrs (per sqft, median)	40.415 (296.908)	16.255
Residential sales past 3 yrs (per sqft, median)	41.073 (270.534)	15.935
Residential sales past 5 yrs (per sqft, median)	44.669 (267.379)	15.571

**Table 2:** Relative risk of demolition (posterior median and 95% credible interval) by crime outcome.

Crime	Demo Level	Low Vacancy	Medium Vacancy	High Vacancy
Violent	low	1.0128 (0.8936, 1.1497)	0.9764 (0.885, 1.0765)	1.0047 (0.9618, 1.0498)
	med	1.0539 (0.8809, 1.2576)	1.0399 (0.8857, 1.2141)	1.0179 (0.9618, 1.076)
	high	1.0759 (0.8554, 1.3422)	1.2301 (1.0106, 1.4815)	1.0657 (1.0017, 1.1348)
Violent w Firearm	low	0.9996 (0.7698, 1.2841)	1.1207 (0.8941, 1.3994)	1.0377 (0.9573, 1.1248)
	med	1.1226 (0.8063, 1.5412)	1.5619 (1.0513, 2.276)	0.9609 (0.8681, 1.0612)
	high	1.0235 (0.6974, 1.4837)	1.0561 (0.5653, 1.831)	1.0156 (0.9086, 1.1356)
Drug	low	0.9487 (0.7946, 1.1295)	1.0767 (0.9336, 1.2405)	1.0186 (0.9548, 1.0853)
	med	1.1647 (0.9125, 1.4805)	0.9432 (0.7246, 1.2147)	1.1399 (1.0524, 1.235)
	high	1.2914 (0.9708, 1.6974)	1.2163 (0.8924, 1.6197)	1.1621 (1.0622, 1.2685)
Vacant Space	low	1.0525 (0.954, 1.1522)	1.0334 (0.9656, 1.1067)	1.01 (0.9794, 1.0421)
	med	1.0477 (0.917, 1.1948)	0.9315 (0.8199, 1.0573)	1.0024 (0.9632, 1.0439)
	high	0.9598 (0.8174, 1.1373)	0.9448 (0.8003, 1.108)	1.0083 (0.9621, 1.0576)
Total	low	1.0188 (0.9427, 1.0987)	1.0241 (0.9668, 1.0852)	1.0089 (0.9827, 1.0356)
	med	1.0378 (0.929, 1.1595)	0.9806 (0.8849, 1.0835)	1.0217 (0.9878, 1.057)
	high	1.0206 (0.8897, 1.1714)	1.0434 (0.9216, 1.1771)	1.0482 (1.0077, 1.0906)

**Table 3:** Relative risk of rehabilitation (posterior median and 95% credible interval) by crime outcome.

Crime	Low Vacancy	Medium Vacancy	High Vacancy
Violent	0.9258 (0.7616, 1.118)	1.0426 (0.9025, 1.1993)	1.0089 (0.9562, 1.0643)
Violent w Firearm	0.9877 (0.6951, 1.3949)	1.275 (0.9059, 1.7422)	1.0102 (0.9086, 1.1219)
Drug	1.0194 (0.8056, 1.2846)	1.247 (1.0018, 1.5416)	0.9692 (0.8944, 1.0483)
Vacant Space	0.9951 (0.8679, 1.142)	0.951 (0.8553, 1.0573)	1.0212 (0.9817, 1.0615)
Total	0.9779 (0.875, 1.0923)	1.003 (0.9216, 1.0903)	1.0084 (0.9765, 1.0417)

## Data Availability

The data used to complete this study are available from ICPSR.

## Appendix A. Data

### Appendix A.1. Imputation

In total, 10 of the 569 hexagons had missing values for the variables mean bedrooms (256 missing values) and mean bathrooms (88 missing values). We conducted multiple imputation using a random forest (RF) imputation algorithm, implemented using the missForest R package, to impute missing data [24,25,32,33]. missForest is a multiple imputation algorithm designed for mixed-type data. Because it is rooted in RF, it performs well even for data with complex characteristics, such as high dimensionality, interactions, and non-linear effects, while maintaining computational efficiency. The iterative algorithm begins by training an RF on the observed data and then using that to predict missing values. This process is repeated multiple times for each variable with missing values until a stopping criterion is met. missForest performs at least as well as competitors, such as k-nearest neighbor imputation, missingness pattern alternating lasso, and multiple imputation with chained equations [32,33]. To estimate accuracy of the imputation, we performed a test case by using complete rows of data and introducing missing values in proportions corresponding to our full dataset. In this simulation, we saw out-of-bag (OOB) error estimates of $5.593\times 10^{-7}$ and true imputation error of 0.0843 (normalized root mean squared error per the package definition). The imputation of our complete data set via missForest showed OOB imputation error of $5.725\times 10^{-7}$.

### Appendix A.2. Crime Variables

Some measures of crime that did not comprise the outcome were used as explanatory variables. For violent crimes, the crime predictors included were burglary, motor vehicle theft, larceny, arson, drug crimes, drunkenness, and vacant space crimes; the same predictors plus rape were used for violent crimes with a firearm. For drug crimes, the crime predictors were burglary, motor vehicle theft, larceny, arson, drunkenness, vacant space crimes, rape, homicide, aggravated assault, and robbery. The crime predictors for vacant space crimes were burglary, motor vehicle theft, drug crimes, drunkenness, rape, homicide, aggravated assault, and robbery.

## Appendix B. Variable Summaries

**Table A1:** Total counts and mean (standard deviation) per hexagon quarter for crime predictors.

Crime	Total	Mean (St Dev)
Aggravated assault	9990.452	0.763 (1.224)
Liquor law violations	692.443	0.081 (0.405)
Arson	1742.745	0.133 (0.409)
Burglary	40,891.375	3.125 (3.379)
Drunkenness	18.000	0.001 (0.037)
Homicide	464.914	0.036 (0.198)
Larceny	83,292.196	6.364 (6.421)
Motor vehicle theft	21,275.205	1.626 (1.875)
Property crimes	147,201.522	11.248 (10.168)
Rape	2579.000	0.197 (0.552)
Robbery	17,100.886	1.307 (1.917)

**Table A2:** Mean and standard deviation of community characteristics by hexagon-quarter.

Variable	Mean (St Dev)
population	646.772 (518.254)
number of employees	210.274 (168.467)
median household income	25,462.860 (15,385.434)
**Percent of hexagon population**	
aged 18–29	16.201 (10.669)
over age 65	12.060 (7.727)
under age 18	19.555 (11.093)
male	42.339 (16.838)
non-Hispanic Black	45.834 (38.022)
Hispanic	8.292 (11.304)
age 25+ with bachelor’s degree	13.408 (13.127)
age 25+ with high school degree	68.667 (27.118)
with managerial job	4.847 (4.737)
below poverty line	31.302 (19.07)
on public assistance	5.676 (4.825)
single parent household	25.602 (15.773)
unemployed	16.110 (11.863)

## Appendix C. Model Coefficients

**Table A3:** Posterior median and 95% credible intervals for coefficients in models for violent crime, violent crime with a firearm, and drug crimes.

	Violent Crime		Violent Crime with Firearm		Drug Crime	
Intercept	−0.0399	(−0.1357, 0.0548)	−1.2729	(−1.4487, −1.0983)	−1.0444	(−1.1771, −0.9139)
Low demo	0.0127	(−0.1125, 0.1395)	−0.0004	(−0.2616, 0.2501)	−0.0527	(−0.2299, 0.1218)
Med demo	0.0525	(−0.1268, 0.2292)	0.1156	(−0.2153, 0.4325)	0.1525	(−0.0916, 0.3924)
High demo	0.0732	(−0.1562, 0.2943)	0.0232	(−0.3605, 0.3945)	0.2558	(−0.0296, 0.5291)
Medium vac	−0.0290	(−0.1296, 0.0737)	0.0439	(−0.1375, 0.2311)	−0.0894	(−0.2208, 0.0438)
High vac	0.0346	(−0.0792, 0.1498)	0.1001	(−0.091, 0.2918)	0.0164	(−0.134, 0.1644)
Rehab	−0.0771	(−0.2723, 0.1116)	−0.0124	(−0.3637, 0.3328)	0.0192	(−0.2162, 0.2504)
Community PC1	−0.2437	(−0.274, −0.2142)	−0.2199	(−0.265, −0.1761)	−0.2509	(−0.2877, −0.2161)
Property PC1	−0.0959	(−0.1202, −0.071)	−0.1069	(−0.1414, -0.0717)	−0.0592	(−0.0889, −0.0293)
Crime PC1	0.0874	(0.0752, 0.0997)	0.1480	(0.1259, 0.1702)	0.1244	(0.1081, 0.1403)
Quarter 1	−0.0910	(−0.1424, −0.0381)	−0.1584	(−0.2806, −0.034)	0.1437	(0.0496, 0.2396)
Quarter 2	0.1315	(0.0784, 0.1848)	0.2309	(0.1061, 0.3521)	0.3424	(0.2433, 0.4432)
Quarter 3	0.2107	(0.1634, 0.2576)	0.2821	(0.172, 0.3895)	0.2270	(0.1393, 0.3151)
Demo, 1 year ago	0.0053	(−0.0027, 0.0131)	0.0015	(−0.0111, 0.0141)	0.0014	(−0.009, 0.0119)
Demo, 2 year ago	−0.0017	(−0.0095, 0.0059)	0.0008	(−0.0117, 0.0134)	0.0052	(−0.005, 0.0156)
Rehab, 1 year ago	−0.0238	(−0.0706, 0.0224)	0.0495	(−0.0345, 0.1304)	−0.0309	(−0.0956, 0.0348)
Rehab, 2 year ago	0.0001	(−0.0496, 0.0497)	0.0093	(−0.0827, 0.1031)	−0.0219	(−0.0937, 0.0482)
Low demo × med vac	−0.0367	(−0.196, 0.1211)	0.1171	(−0.2211, 0.4534)	0.1268	(−0.0921, 0.3534)
Med demo × med vac	−0.0141	(−0.2498, 0.2238)	0.3341	(−0.1736, 0.8263)	-0.2113	(−0.5579, 0.1399)
High demo × med vac	0.1337	(−0.1604, 0.4283)	0.0322	(−0.6951, 0.714)	−0.0592	(−0.4662, 0.3415)
Low demo × high vac	−0.0081	(−0.1403, 0.123)	0.0369	(−0.2229, 0.3099)	0.0719	(−0.1126, 0.2588)
Med demo × high vac	−0.0350	(−0.2164, 0.1502)	−0.1554	(−0.4799, 0.1854)	−0.0222	(−0.2671, 0.2338)
High demo × high vac	−0.0093	(−0.241, 0.2255)	−0.0095	(−0.3877, 0.3898)	−0.1069	(−0.3901, 0.1872)
Med vac × rehab	0.1199	(−0.113, 0.3581)	0.2558	(−0.2363, 0.7308)	0.2015	(−0.1141, 0.5148)
High vac × rehab	0.0857	(−0.107, 0.2871)	0.0229	(−0.3377, 0.3877)	−0.0509	(−0.2916, 0.1928)

**Table A4:** Posterior median and 95% credible intervals for coefficients in models for drug crime and vacant space crime.

	Vacant Space Crime		Total Crime	
Low demo	0.0511	(−0.0471, 0.1417)	0.0186	(−0.0591, 0.0941)
Med demo	0.0466	(−0.0866, 0.178)	0.0371	(−0.0736, 0.148)
High demo	−0.0411	(−0.2017, 0.1287)	0.0203	(−0.1169, 0.1582)
Med vac	0.0714	(−0.0052, 0.149)	−0.0044	(−0.075, 0.0635)
High vac	0.1207	(0.0351, 0.208)	0.0551	(−0.0221, 0.1327)
Rehab	−0.0050	(−0.1417, 0.1327)	−0.0224	(−0.1336, 0.0883)
Community PC1	−0.2065	(−0.2297, −0.1841)	−0.2302	(−0.2516, −0.2086)
Property PC1	−0.1058	(−0.1256, −0.087)	−0.1112	(−0.1292, −0.093)
Crime PC1	0.0441	(0.0348, 0.0538)	NA	NA
Quarter 1	−0.0994	(−0.1661, −0.0328)	−0.0926	(−0.1429, −0.042)
Quarter 2	0.1144	(0.045, 0.1856)	0.0936	(0.0389, 0.1467)
Quarter 3	0.1870	(0.1267, 0.2511)	0.1744	(0.1269, 0.2203)
Demo, 1 year ago	−0.0003	(−0.0067, 0.0062)	0.0015	(−0.0043, 0.0073)
Demo, 2 year ago	0.0017	(−0.0044, 0.008)	0.0004	(−0.005, 0.0062)
Rehab, 1 year ago	0.0251	(−0.0109, 0.0612)	0.0008	(−0.0314, 0.0327)
Rehab, 2 year ago	−0.0138	(−0.0533, 0.0248)	−0.0085	(−0.0419, 0.0253)
Low demo × med vac	−0.0185	(−0.1307, 0.1007)	0.0059	(−0.0921, 0.0997)
Med demo × med vac	−0.1211	(−0.2999, 0.0713)	−0.0564	(−0.2091, 0.0919)
High demo × med vac	−0.0156	(−0.2511, 0.2083)	0.0221	(−0.1626, 0.2041)
Low demo × high vac	−0.0410	(−0.1375, 0.0631)	−0.0096	(−0.0895, 0.0712)
Med demo × high vac	−0.0447	(−0.1847, 0.0963)	−0.0153	(−0.1312, 0.1)
High demo × high vac	0.0493	(−0.1223, 0.2176)	0.0262	(−0.117, 0.1679)
Med vac × rehab	−0.0441	(−0.2178, 0.1252)	0.0249	(−0.113, 0.1629)
High vac × rehab	0.0264	(−0.1131, 0.1672)	0.0304	(−0.0855, 0.1439)
